# Internet-Based Cognitive Behavioral Therapy Interventions for Caregivers of Patients With Cancer: Scoping Review

**DOI:** 10.2196/67131

**Published:** 2025-06-04

**Authors:** Chun Tong Shen, Jian Shi, Feng Xia Liu, Xiao Meng Lu

**Affiliations:** 1 Department of Oncology, The Fourth Hospital of Hebei Medical University, No. 12 Health Road, Shi Jiazhuang, 050000, China, 86 0311-86296362, 86 13831110729; 2 Department of Nursing, The Fourth Hospital of Hebei Medical University, Shi Jiazhuang, China; 3 Department of Radiotherapy, The Fourth Hospital of Hebei Medical University, Shi Jiazhuang, China

**Keywords:** cancer, oncology, caregivers, informal caregivers, internet, scoping review, cognitive behavioral therapy

## Abstract

**Background:**

Cancer imposes significant physical and emotional distress not only on patients, but also on their caregivers. In recent years, there has been a growing focus on the mental and physical well-being of caregivers. Among various psychological interventions, cognitive behavioral therapy (CBT) is widely recognized as one of the most effective approaches. However, traditional CBT is often limited by time and geographical constraints, resulting in delayed or inefficient support for caregivers. Internet-based cognitive behavioral therapy (ICBT) presents a valuable alternative for alleviating the caregiving burden and the negative emotions experienced by caregivers.

**Objectives:**

This study aimed to provide a scoping review of ICBT interventions for caregivers of patients with cancer, examining intervention content, outcome measures, and effectiveness and to offer insights and references for the development and clinical applications of ICBT programs tailored to caregivers of patients with cancer in China.

**Methods:**

Relevant literature was systematically searched in PubMed, Web of Science, Cochrane Library, CINAHL, Embase, China National Knowledge Infrastructure (CNKI), Wanfang Data, and VIP Chinese Journal Database. The search timeframe was from database inception to June 6, 2024. Inclusion criteria encompassed intervention studies that implemented cognitive behavioral therapy for caregivers of patients with cancer via the internet, WeChat (Tencent), or mobile electronic devices. This category includes both randomized and nonrandomized controlled trials.

**Results:**

A total of 12 studies met the criteria and were included in the review. The intervention content included the following components: treatment initiation and brief introduction (5/12, 41%), cognitive education and restructuring (7/12, 58%), emotional expression and coping (6/12, 50%), cognitive restructuring and reinforcement (4/12, 33%), behavioral training and activation (9/12, 75%), problem-solving techniques (4/12, 33%), communication (5/12, 41%), and completion of treatment with follow-up consolidation (3/12, 25%). The intervention duration typically ranged from 6 to 8 weeks. Outcome indicators encompassed feasibility and acceptability, anxiety, depression, caregiver burden, and quality of life. ICBT demonstrated positive effects for caregivers of patients with cancer. Most intervention programs were feasible and acceptable, with 2 out of 5 feasibility studies reporting recruitment rates below 50%. Attrition rates across studies ranged from 3% to 16%, and caregivers expressed satisfaction with the information, quality, and skills provided. ICBT exhibits a moderate effect in diminishing negative emotions among caregivers and alleviating caregiver stress. However, its impact on improving quality of life is not statistically significant, underscoring the need for long-term follow-up.

**Conclusions:**

The implementation of ICBT for caregivers of patients with cancer has demonstrated beneficial outcomes, attributed to its practicality and flexibility, which contribute to its greater acceptance among caregivers. Nevertheless, there is significant heterogeneity in intervention format, duration, and outcome indicators. It is necessary to develop optimal intervention strategies and secure online platforms based on the cultural background in China to improve the quality of life of caregivers.

## Introduction

The incidence and mortality rates of cancer are rapidly increasing globally. According to the International Agency for Research on Cancer, there were 19.29 million new cancer cases and 9.96 million cancer deaths worldwide in 2020, and the rise in cancer incidence and mortality rates has resulted in a significant disease burden on people [1]. The diagnosis and long-term treatment of cancer not only cause adversity for patients but also impose psychological stress and burdens on caregivers [2]. Caregivers of patients with cancer refer to informal caregivers, including family members, partners, or friends. They provide unpaid social, emotional, and economic support to a family member with cancer requiring care and are involved throughout the patient’s symptom management and nursing [3,4]. Caregivers attend to the daily needs of patients and fulfill family responsibilities; they also serve as the patient’s primary emotional support. Due to complex treatment environments, a lack of disease-related knowledge, and significant economic burdens, caregivers often experience negative emotions such as anxiety and depression [5]. Zhou et al [6] found that 60.7% of caregivers of patients with cancer experience sleep disturbances. Yang et al [7] conducted a survey involving 116 caregivers of terminally ill patients with cancer receiving home care, revealing that 83.62% of the caregivers reported experiencing moderate to severe fatigue, primarily characterized by physical fatigue. Geng et al [8] reported that the prevalence of anxiety and depression among caregivers of patients with cancer was 46.55% and 42.30%, respectively, with 62% of caregivers bearing a heavy burden that negatively affected their daily lives. Therefore, attention should be given to the physical and mental health of caregivers, along with the provision of appropriate supportive care.

Current interventions for caregivers of patients with cancer include psychosocial support, education, and informational support [9]. Cognitive behavioral therapy (CBT) has received considerable attention owing to its robust theoretical foundation, brief treatment duration, and well-defined structural approach. However, traditional CBT is often influenced by economics, time, and spatial factors, preventing some caregivers from accessing effective help and support [10]. In recent years, with the rise of the “Internet+Healthcare” service model, internet-based cognitive behavioral therapy (ICBT) has emerged. ICBT is an internet-based treatment approach that uses tools such as computers and mobile devices to deliver the core content and skills of CBT through text, video, images, and audio [11].

ICBT addresses the limitations of CBT in its application. Some caregivers concentrate on caregiving behaviors, frequently suppressing their own emotions, which may lead to distress stemming from a deficiency in caregiving skills. ICBT provides caregivers with a discreet online platform that allows them to access relevant information at any time through simple and user-friendly self-service methods, facilitating timely communication with health care professionals and enhancing their cognitive abilities. In addition, techniques such as emotional guidance and relaxation training are used to alleviate caregiver stress and improve their quality of life (QoL) [12,13]. Existing studies have shown that ICBT can mitigate the anticipatory grief experienced by caregivers of patients with cancer, decrease caregiving burden, and improve their self-efficacy [14].

Currently, research on the application of ICBT for caregivers of patients with cancer is steadily growing. However, there is significant heterogeneity in the forms of online interventions, intervention content, and outcome indicators. To gain a comprehensive understanding of the current research status of ICBT, this study uses a scoping review to systematically analyze pertinent studies from both domestic and international contexts. Our goal is to provide references to promote the use and dissemination of ICBT among caregivers of patients with cancer in China.

## Methods

### Study Design and Framework

This scoping review adhered to the methodological framework developed by the Joanna Briggs Institute (JBI; 2019) [15]. The reporting follows the PRISMA-ScR (Preferred Reporting Items for Systematic Reviews and Meta-Analyses Extension for Scoping Reviews) guidelines (Checklist 1).

### Research Questions

The review addressed three key questions: (1) what are the intervention components of ICBT for caregivers of patients with cancer?; (2) what are the intervention forms, duration, and evaluation time points for ICBT?; and (3) what are the outcome indicators and effects of ICBT interventions?

### Search Strategy

A systematic search was conducted across 9 databases, including CNKI, Wanfang Database, China Biomedical Literature Database, VIP, PubMed, Web of Science, Embase, CINAHL, and Cochrane Library. The search timeframe extended from the establishment of the databases to June 6, 2024. A combination of subject headings and free-text terms was used. The search strategy was formulated with the guidance of a librarian. The English search terms were “neoplas*, carcinoma*, tumor, oncology, cancer*;” “Cognitive Behavio*, Behavio* Therap*, Cognitive Therap*, ICBT, cognitive behavioural therapy, CCBT;” “online, network, Internet, smartphone, telephone, computer;” and “caregiver*, spouse, family, informal caregiver, couple*.” The search strategy for each database was documented in the Multimedia Appendix 1.

### Study Selection

The eligibility criteria is presented in Textbox 1.

Textbox 1. **Inclusion and exclusion criteria for article selection.**

**Inclusion criteria:**

- Study participants: caregivers of confirmed (by pathology or imaging) patients with cancer, including offspring, parents, and spouses, aged 18 years or older.
- The intervention emphasizes the implementation of cognitive behavioral therapy via the internet, WeChat, mobile devices, or other applications.
- Literature type: original research, including randomized controlled trials or quasiexperimental studies.
- Published literature in both Chinese and English.

**Exclusion criteria:**

- Literature for which the full text could not be obtained.
- Duplicated publications.
- Conference abstracts.
- Research protocols, reviews, and case studies.

### Data Extraction and Quality Assessment

The literature search results were imported into EndNote X9 for duplicate removal. Two independent reviewers (CTS) and (XML) screened titles, abstracts, and full texts against the predefined inclusion and exclusion criteria. Discrepancies were resolved through discussion with a third researcher to reach consensus. One reviewer (CTS) extracted study data using a standardized Microsoft Excel form, capturing authors, publication year, country, design, population characteristics, sample size, interventions, and outcomes, with a second reviewer (XML) independently verifying the accuracy and completeness of all extracted data. For included randomized controlled trials, we conducted quality assessments using the Cochrane Risk of Bias Tool (version 5.1.0) [16], categorizing studies as grade A (low risk), B (moderate risk), or C (high risk), with any discrepancies resolved through consultation with a third researcher to reach consensus.

## Results

### Study Selection and Characteristics

**Figure 1.**
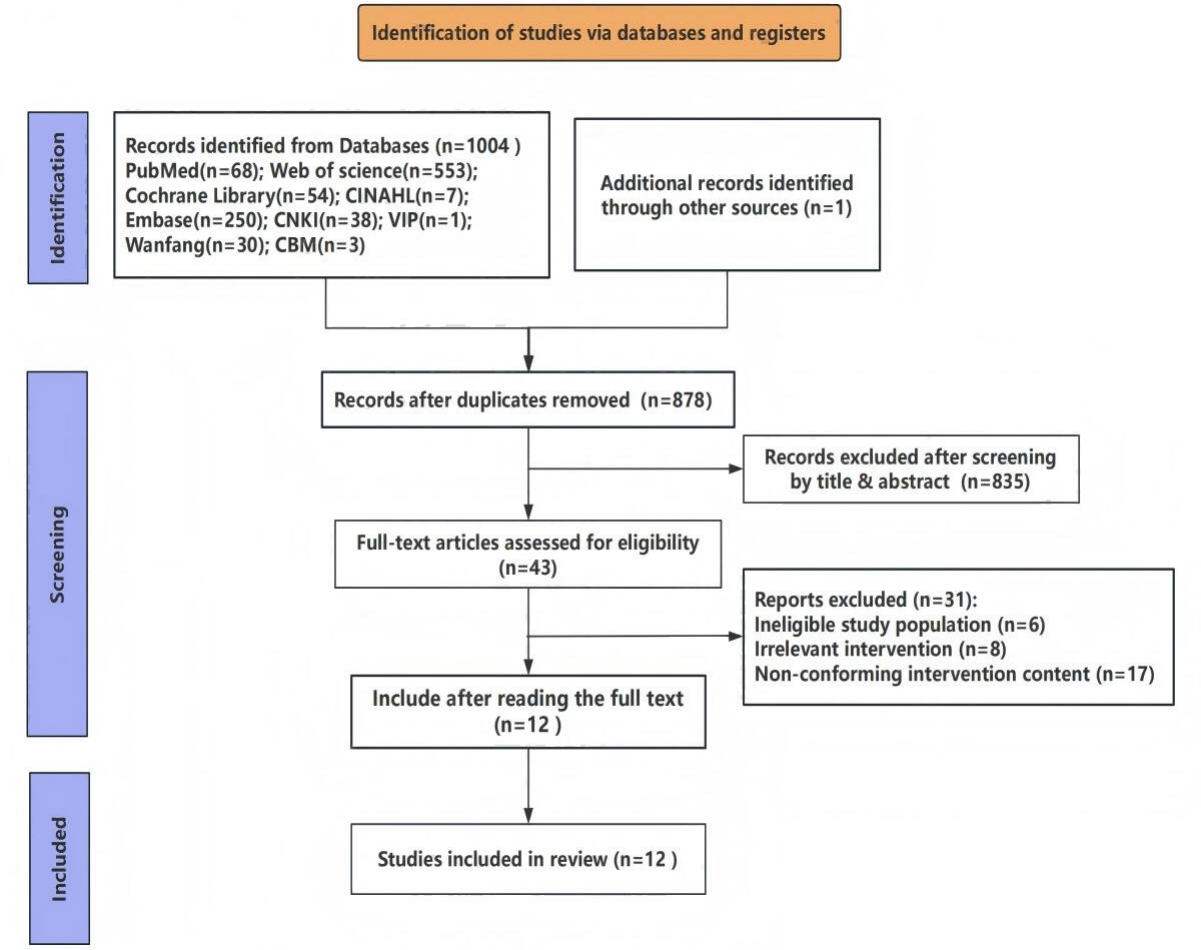
Literature screening process diagram.

The systematic search identified 1005 records, with 12 studies meeting inclusion criteria after screening (Figure 1) [14,16-26]. The studies (2013‐2023) represented diverse geographic regions, such as the United States (4/12, 33%), Australia (3/12, 25%), China (3/12, 25%), Lithuania (1/12, 8%), and Germany (1/12, 8%). Study designs included randomized controlled trials (9/12, 75%), quasi-experimental (1/12, 8%), mixed-methods (1/12, 8%), and feasibility studies (1/12, 8%). Quality assessment of randomized controlled trials (RCTs) indicated moderate methodological rigor (B-level). Table 1 details the basic characteristics of the included studies.

**Table 1.** Basic characteristics of included literature.

Reference	Study type	Interference objects	Sample (example, T/C)	Intervention and groups	Outcome indicators; evaluation time	Results
Scott et al [17]	Feasibility of the intervention	Carers of adult patients with cancer receiving curative treatment	13	Therapist-administered 6-week ICBT^a^ program via website and email. CCO^b^ components included: starting treatment; coping with physical symptoms and side effects; coping with emotional distress; body image, identity and sexuality; family and friends; completing treatment.	Primary outcomes: feasibility and acceptability; secondary outcomes: negative affect, distress, QoL; 2-time point evaluation (pre-post)	Engagement was modest; CCO resulted in large overall reductions in negative affect (Cohen *d*=0.88) and small reductions in cancer-specific distress (Cohen *d*=0.37), small to moderate increases in QoL.
Chambers et al [18]	RCT^d^	Patients with cancer and caregivers	345/345	A 5-session psychologist cognitive-behavioral intervention delivered by telephone (psychoeducation; coping and stress management skills; problem solving; cognitive therapy; enhancing support networks). A single session of nurse-led self-management intervention.	Primary outcomes: psychological; cancer-specific distress and posttraumatic growth; 4-time point evaluation (pre-3-6-12 months).	The psychologist-led intervention demonstrated reductions both psychological distress (Cohen *d*=0.2, *P*<.001) and cancer-specific distress (Cohen *d*=0.77, *P*<.001), while also enhancing positive adjustment (Cohen *d*=0.82, *P*<.001) from baseline to 12 months.
Mosher et al [19]	RCT	Patients with lung cancer and their family caregivers	51 pairs/55 pairs	Psychologists and clinical social workers delivered the TSM^e^ intervention to participants via telephone, with 4 weekly sessions. TSM components included: relaxation; cognitive restructuring; problem-solving; self-soothing/emotion-focused approach; pleasant activities; activity pacing; communication; plan for continued skills practice. Education or support	Primary outcomes: depression and anxiety; secondary outcomes: self-efficacy and caregiver burden; 3-time point evaluation (baseline-2‐6 weeks postintervention).	Small effects in favor of TSM were found regarding caregiver self-efficacy for managing their own emotions and perceived social constraints from the patient.
Kubo et al [20]	2-arm RCT	Patients undergoing cancer chemotherapy and caregivers	Patients: 54/43; caregivers: 17/14	Psychologists, psychosocial workers, and nurses implemented the 8-week Headspace program through website or mobile apps. Headspace: encourage participants to first complete a 30-day mindfulness meditation foundation course; they can also choose 10 to 30 days of related symptom or meditation courses. UC^f^	Primary outcomes: mindfulness and quality of life; secondary outcomes: distress; posttraumatic growth; fatigue; sleep quality; 2-time point evaluation (pre-post).	Headspace significantly improved mindfulness (*P*=.03) with borderline significant effects on PTGI^g^ new possibilities (*P*=.06) versus controls
Biliunaite et al [21]	2-arm RCT	Caregivers of individuals with dementia, cancer, or other illnesses	31/32	Therapists implemented an 8-week ICBT program through the Slaugau Artima website. ICBT: introduction; thoughts; stress and relaxation; problem solving; communication; anxiety; behavioral activation; and maintenance. UC	Primary outcome: CBI^h^; secondary outcomes: depression, anxiety, stress, and QoL; 2-time point evaluation (pre-post).	ICBT showed large effects on burden reduction (*P*<.001) and stress (*P*<.001), moderate effects on anxiety (*P*=.004) and depression (*P*=.01), and significant QoL enhancement (*P*=.001).
Luo et al [22]	RCT	Parents of children diagnosed with cancer	52/51	Psychologists, doctors, and nurses implemented the 8-week a mobile device–based resilience training program. Resilience training program: understanding the purpose of intervention, relaxation technique training, problem-solving skills, cognitive restructuring, promoting good relationships, and cultivating positive performance and beliefs, etc. UC	Primary outcome: resilience; secondary outcomes: depressive symptoms and QoL; 3-time point evaluation (pretreatment, 2 and 6 months after the intervention began).	At the 6-month follow-up, the intervention demonstrated statistically significant improvements in resilience (*P*=.01) and depressive symptoms (*P*=.04), but failed to show significant QoL enhancement (*P*=.38), although the experimental group showed numerically higher QoL scores than controls.
Wakefield et al [23]	3-arm RCT	Parents or caregivers of children	19/18/19	Psychologist-delivered online intervention. Cascade: introduction and behavioral activation; identifying and challenging unhelpful thoughts; mindfulness and disengagement; skills for fostering relationships and living a rich life after cancer; booster session. Peer-support or waitlist.	Feasibility; acceptability; safety; efficacy (QoL; psychological outcomes); 4-time point evaluation (baseline, 2‐4 weeks post-intervention; 2‐4 weeks post-booster and 6 months postintervention).	Most Cascade parents were satisfied and reported experiencing benefits from the program. However, Cascade did not improve their main outcomes, including parents’ quality of life, depression and anxiety.
Trevino et al [24]	RCT	Older adults with cancer and their caregivers	14 pairs/14 pairs	Social workers provide 7-session over the telephone. MAC^i^: this includes providing information on treatment methods for the elderly, addressing the widespread shame associated with psychological services among the elderly, and integrating strategies to address cancer-specific stressors during the intervention process. UC	Feasibility, acceptability, participant adherence, anxiety, depression, and QoL; 2-time point evaluation (pre-post).	85.7% participants completed all 7 sessions, over 80% of caregivers rated MAC as “moderately” to “very” helpful. MAC dyads experienced a greater reduction in anxiety than dyads in usual care with smaller changes in depression and quality of life.
Kaiser et al [25]	RCT	Caregivers of cancer bereavement	44/43	Caregivers completed 2 self-scheduled 45-minute writing sessions weekly via a website. ICBT: 10 structured writing tasks, 3 modules (self-confrontation; cognitive reappraisal; and social sharing). Waitlist	Primary outcome: prolonged grief; secondary outcomes: depression, anxiety, posttraumatic stress, posttraumatic growth, somatization, sleep quality, and mental and physical health; 5-time point evaluation (baseline, posttreatment, and 3‐6-12 months).	ICBT reduced symptoms of prolonged grief (Cohen *d*=0.80; *P*<.001) to a clinically significant extent. It had favorable effects on depression, anxiety, posttraumatic stress, posttraumatic growth, and overall mental health but not on somatization, sleep quality, or physical health.
Yang et al [26]	RCT	Patients undergoing cervical cancer chemotherapy and their spouses	53 pairs/53 pairs	Psychological counselors and nurses delivered an 8-week ICBT program through WeChat (Tencent) public platform and offline group sessions. ICBT: psychological diagnosis; cognitive education; behavior training; emotional expression; consolidate follow-up. UC	The patients’ distress and QoL; 2-time point evaluation (pre-1 month after intervention).	ICBT significantly reduced the detection rate of psychological distress (*P*<.05) and improved quality of life (*P*<.01) among patients.
Carr et al [27]	Mixed research	Caregivers of a phase 1 oncology trial patient	23 quantitative cases; 5 qualitative cases	Clinician-administered telephone intervention comprised 4 CBMS^j^+4 randomized CBT sessions across 9 weeks. CBSM: mind-body connection; coping skills; communication; and social support; metta meditation. CBT: intro to CBT-tracking automatic thoughts; identifying distorted thoughts; challenging distorted thoughts; core beliefs or relapse prevention	Primary outcome: acceptability and feasibility; secondary outcomes: caregiver distress; caregiver burden; PAC^k^; caregiver grief; anxiety and depression; 2-time point evaluation (pre-post).	Although the P1CaLL^l^ pilot achieved limited recruitment feasibility (45.3% enrollment), its high acceptability (84% completion) and preliminary efficacy signals across multiple caregiver outcomes (stress reduction, isolation mitigation, and self-control improvement) support further development.
Wang [14]	Quasiexperimental study	Caregivers of patients with cancer	38/38	Physicians and nurses administered ICBT through a website and WeChat groups, including twice-weekly digital content and monthly peer support video sessions. ICBT: basic knowledge; symptom education; common knowledge of home care; relaxation training; social support UC	Care burden, anticipatory grief, and self-efficacy; 3-time point evaluation (pretreatment, posttreatment 6-12 weeks).	ICBT demonstrated significant efficacy in reducing caregivers’ anticipatory grief levels (*P*<.001), alleviating caregiving burden (*P*<.001), and enhancing self-efficacy (*P*<.001).

a ICBT: internet-based cognitive behavioral therapy.

b CCO: cancer coping online.

c QoL: quality of life.

d RCT: randomized controlled trial.

e TSM: telephone-based symptom management.

f UC: usual care.

g PTGI: posttraumatic growth inventory.

h CBI: caregiver burden inventory.

i MAC: managing anxiety from cancer.

j CBSM: cognitive behavioral stress-management.

k PAC: positive aspects of caregiving.

l P1CaLL: Phase 1 Caregiver LifeLine.

### Intervention Content

The included studies incorporated the following key components in their ICBT interventions for caregivers (Textbox 2).

Textbox 2. **Key components of internet-based cognitive behavioral therapy interventions for caregivers.**

- Treatment introduction: a total of 5 studies [14,17,21,23,26] initiated interventions with an introductory phase, wherein researchers presented the medical center and care team to establish caregiver-provider collaboration. Before intervention delivery, caregivers underwent baseline assessments, received psychoeducation on cognitive-behavioral therapy principles, and were guided on study protocols to optimize adherence. In addition, they were encouraged to set personalized goals to enhance engagement and motivation.
- Cognitive education and restructuring: a total of 7 studies [14,18,19,22,24-26] incorporated structured modules to help caregivers reframe maladaptive cognitions. Through perspective-taking exercises and peer-sharing sessions, participants were educated on disease-related knowledge to foster accurate perceptions of cancer. This component also emphasized symptom recognition training to improve timely and appropriate caregiving responses.
- Emotional expression and coping: a total of 6 studies [17-19,21,26,27] assessed caregivers’ emotional states using standardized questionnaires or individual interviews, followed by discussions on psychosocial impacts. To mitigate distress, interventions introduced techniques such as cognitive restructuring diaries, mindfulness meditation, and progressive relaxation training.
- Cognitive restructuring and reinforcement: a total of 4 studies [22,23,25,27] focused on identifying and modifying maladaptive thought patterns. Caregivers were taught to recognize automatic thoughts and common cognitive distortions. Through guided exercises, they practiced challenging unhelpful beliefs to cultivate healthier cognitive frameworks.
- Behavioral training and activation: the most frequently implemented component (9 studies [14,17,19-24,26]) involved skill-building through evidence-based techniques such as progressive muscle relaxation, diaphragmatic breathing, music therapy, and guided imagery. Caregivers selected preferred modalities based on individual capacity and preferences. Protocols also emphasized self-care strategies, including scheduled personal time and reward systems.
- Problem-solving techniques: a total of 4 studies [18,19,21,22] trained caregivers in structured problem-solving. Participants learned to deconstruct challenges, identify barriers, evaluate coping strategies, and implement solutions. Postintervention, they monitored outcomes and adjusted approaches through reflective practice to enhance long-term adaptive skills.
- Communication: a total of 5 studies [17,19,21,23,27] addressed communication dynamics, exploring how caregiving roles influenced interpersonal interactions. Caregivers practiced maintaining or improving intimacy through verbal and nonverbal techniques, sustaining social connections, and fostering supportive relationships.
- Completion of treatment and consolidation follow-up: a total of 3 studies [17,21,26] concluded with a review phase, summarizing key concepts and reinforcing long-term skill retention. Caregivers were guided in self-directed practice, goal reflection, and future planning to sustain intervention benefits long-term skill retention. Caregivers were guided in self-directed practice, goal reflection, and future planning to sustain intervention benefits.

The included studies typically incorporated 3‐5 core intervention modules. Following each intervention session, participants were required to complete structured homework assignments, which included documenting caregiving-related emotional experiences, evaluating automatic thoughts using thought records, emotional expression through writing exercises [21], completion of structured writing tasks [25], and home practice of acquired skills [19,20].

The reviewed literature revealed 2 distinct ICBT delivery models for caregivers. Seven studies [14,17,21-23,25,27] implemented caregiver-specific interventions focusing on cognitive restructuring, emotional regulation training, and evidence-based relaxation techniques to enhance multidimensional wellbeing. Alternatively, 5 studies [18-20,24,26] used dyadic approaches that simultaneously engaged both patients and caregivers through adapted protocols delivered in either parallel or joint therapeutic sessions. Both models demonstrated effectiveness in addressing the psychological needs of caregivers while accounting for different caregiving contexts.

### Intervention Elements

The ICBT interventions examined in this study comprised several key components, such as delivery modality, provider qualifications, intervention duration, and evaluation timelines. The primary delivery modalities included web-based platforms (4/7, 57%) studies with real-time psychologist support or email feedback [14,17,20-22,25,26], telephone sessions (45‐60 min) [18,19,24,27], and video conferencing [23]. Interventions were predominantly delivered by psychotherapists, with 1 study [22] using a multidisciplinary team (psychologists, physicians, and nurses). Intervention duration varied (most commonly 6‐8 weeks [17,20-22,26]) depending on content and format. Telephone sessions typically lasted 45‐60 minutes [19,24], though some flexible protocols permitted completion within 1 week [14,17,21,26]. Effectiveness was evaluated at 3 time points: (1) baseline (preintervention, confirming group comparability [*P*>.05]), (2) postintervention, and (3) follow-up (1‐12 months) in 4 studies [18,22,23,25]. Qualitative components (semistructured interviews) were included in 2 studies [17,27] to assess participant experiences.

### Outcome Indicators and Effectiveness Evaluation of Interventions

The ICBT interventions evaluated 7 primary outcome measures: feasibility (n=4 studies [17,23,24,27]), acceptability (n=4 studies [17,23,24,27]), QoL (n=8 studies [17,20-26]), caregiver burden (n=4 studies [14,19,21,27]), psychological distress (n=11 studies [14,17-25,27]), posttraumatic growth (n=3 studies [18,20,25]), and self-efficacy (n=3 studies [14,19,23]).

#### Feasibility

Feasibility was assessed based on recruitment, retention, and completion rates. In total, 2 studies [17,27] reported recruitment rates below 50%, primarily attributed to participants’ aversion to online support, lack of interest, demanding work schedules, and substantial caregiving commitments. Although most participants completed all intervention modules, attrition occurred due to heightened psychological distress, time constraints, or deterioration of the care recipient’s health [20]. Attrition rates generally ranged from 3% to 16%, with one exception reaching 31% [17]. Qualitative analysis suggests this discontinuation pattern may result from both the lack of personalized engagement in digital interventions and deliberate withdrawal after achieving therapeutic objectives.

#### Acceptability

Acceptability was assessed through participant-reported satisfaction with both intervention content and engagement modalities. Many caregivers expressed positive feedback regarding the intervention, stating that “the online intervention is convenient, time-saving, and practical,” “they were satisfied with the information and quality provided,” “the skills learned were relevant to cancer treatment,” and “the intervention courses helped alleviate stress.” However, 1 study [17] indicated that 33% of participants felt the intervention did not adequately address the needs of caregivers, while another study revealed disagreement about whether patients and caregivers should be treated together. Specifically, 45% of caregivers preferred individual interventions, 36.4% favored some combined treatment, and 18.2% preferred fully integrated interventions.

#### Psychological Outcomes of Caregiver Intervention

Cognitive-behavioral interventions demonstrated measurable benefits for caregiver mental health. The stress management program by Carr et al [27] significantly improved caregivers’ stress coping abilities (effect size *r*=0.39), while 2 other trials [14,21] reported statistically significant reductions in caregiver burden (*P*<.05). Caregivers often experience negative emotions such as anxiety, depression, or sadness due to prolonged caregiving and stress [28]. ICBT interventions showed moderate efficacy in alleviating anxiety and depression [17,21], with the structured writing intervention (“Online-Trauertherapie”) by Kaiser et al [25] producing effect sizes ranging from 0.29 to 0.84 across multiple psychological domains. Notably, Trevino et al [24] found patient-caregiver anxiety changes were positively correlated, though other psychological outcomes showed nonsignificant associations. Some studies [23,29] reported limited effects on anxiety or depression, potentially due to low baseline distress levels or requiring specific intervention components (eg, guided imagery) to achieve psychological benefits.

#### QoL

The included studies reported mixed effects on caregiver’s QoL. A total of 4 trials [17,20,21,25] demonstrated statistically significant QoL improvements following intervention. Notably, one spouse-focused intervention [26] showed significant patient QoL benefits, suggesting potential secondary effects. However, 3 studies [22-24] found no significant QoL changes for caregivers, potentially due to shorter intervention durations or differing outcome measures.

## Discussion

### Principal Findings and Comparison With Previous Work

This study examines the existing research on ICBT interventions for caregivers of patients with cancer. A systematic review of 12 eligible studies revealed that ICBT is a feasible and acceptable approach for this population. The findings suggest that ICBT may alleviate anxiety and depressive symptoms among caregivers, with some studies additionally reporting reduced caregiver burden and enhanced self-efficacy.

These findings are consistent with previous studies, demonstrating the acceptability and feasibility of ICBT when applied to caregivers. For instance, Meichsner et al [30] observed high satisfaction and enhanced well-being among dementia caregivers following ICBT. Similarly, Tur et al [31] documented strong participant satisfaction in an ICBT program for prolonged grief disorder, with 75% of participants achieving clinically significant reductions in depressive symptoms and 50% demonstrating meaningful improvements in grief-related cognitions. Further corroborating these results, Titov et al [32] reported that 63% of individuals with generalized anxiety disorder experienced significant anxiety reduction after ICBT, with concurrent improvements in comorbid depression.

However, the effects of ICBT on caregivers’ QoL were inconsistent across studies. While some improvement in QoL was observed, the overall effect was not statistically significant, possibly due to the relatively short intervention duration. Although follow-up assessments were conducted in some studies, no significant differences in QoL were detected. Li et al [33] suggested that improvements in QoL generally require a longer time to manifest compared with behavioral or mental health changes. In addition, multiple factors, such as caregivers’ socioeconomic status, age, caregiving duration, coping strategies, the patient’s clinical condition, and available social support may influence intervention outcomes [34]. Therefore, future studies should implement multidimensional assessments of caregiver well-being (addressing physical, psychological, emotional, and social domains) to inform personalized interventions, while extending intervention durations to better evaluate long-term QoL outcomes.

This scoping review synthesizes existing literature on ICBT interventions for caregivers of patients with cancer, offering key insights into the current evidence base. Our findings highlight the feasibility and acceptability of most programs; however, recruitment challenges were evident, with a substantial proportion of eligible caregivers declining participation. Furthermore, enrolled participants exhibited high attrition rates. To enhance intervention engagement and retention, future studies should focus on precise population targeting, refined inclusion criteria, and proactive baseline assessments to identify and support at-risk caregivers.

### Population Specificity and Methodological Variability

Among the included studies, 3 investigations [21,24,25] specifically targeted subgroups with distinct characteristics, such as caregivers exhibiting elevated anxiety levels or heightened caregiving burdens. Another 3 studies [19,26,27] enhanced research specificity by focusing on particular cancer types. Several studies treated patient-caregiver dyads as intervention units [24,26], demonstrating that caregiver support could reduce patients’ psychological distress and improve their QoL. This reciprocal relationship reflects how caregivers’ comprehensive support impacts patients’ recovery and emotional state, while patients’ conditions similarly affect caregivers’ wellbeing. Li et al [35] found dyadic collaboration improved both QoL and coping outcomes, though some caregivers preferred individual interventions. Future research should systematically compare the efficacy of caregiver-only interventions versus dyadic intervention approaches.

In total, 2 studies [17,27] included qualitative postintervention evaluations. Carr et al [27] reported that caregivers benefited from recognizing automatic thought patterns, which improved their awareness of irrational thinking and overall wellbeing. However, participants suggested improvements, including initial in-person contact with facilitators, more personalized resources, and better scheduling to accommodate caregiving duties. While ICBT applications for caregivers remain exploratory, mixed-methods approaches combining quantitative outcome measures with qualitative insights can strengthen the evidence base for optimizing interventions [36].

### Exploring Intervention Content and Delivery Formats

Effective intervention design must prioritize both engagement and usability through personalized support programs that address caregivers’ specific needs. A critical yet frequently overlooked component is the implementation of preparatory phases, as evidenced by our review finding that only 5 studies incorporated preintervention assessments. These preliminary modules should systematically collect caregiver characteristics to inform tailored recommendations, which could significantly improve both participation rates and intervention adherence. Establishing such foundational elements represents an important direction for future research development.

The caregiving role often generates substantial psychological burdens, stemming from challenging role transitions, difficulties adapting to medical environments, and the inherent stress of managing a loved one’s illness. Compounding these issues, caregivers frequently suppress their emotions and neglect self-care, resulting in diminished QoL [37,38]. Research by Zhang et al [39] demonstrates that targeted interventions incorporating relaxation training can yield multiple benefits, such as regulating neuropsychological functions, alleviating chronic physical tension, improving physiological responses, and reducing illness-related stress, ultimately decreasing caregiver burden while promoting positive behavioral changes. Therefore, intervention content should integrate 3 key components: (1) cognitive restructuring to modify maladaptive thought patterns; (2) emotional support systems; and (3) practical strategies including context-appropriate health education and relaxation techniques. This comprehensive approach will better equip caregivers to manage their multifaceted challenges.

This study found that few interventions target caregivers’ self-symptom management. While 2 studies assessed interventions for caregiver symptoms (eg, pain, fatigue, and sleep) [20,25], no significant improvements were observed, possibly due to differing intervention focuses. However, Shaffer et al [40] reported substantial reductions in insomnia among cancer caregivers through an internet-based program, even though it was not caregiver-specific. Similarly, Ye et al [41] demonstrated that ICBT improved sleep, anxiety, and depression in patients with insomnia . Future research should develop symptom-specific interventions tailored to caregivers to validate their efficacy.

Our study found that online caregiver interventions typically involve real-time psychologist support or email feedback. To optimize website- or WeChat-based interventions, integrating interactive features (eg, chat functions and message boards) is recommended. Caregivers should receive prompt module-specific feedback via email, with professionals addressing inquiries swiftly to build trust. Evidence confirms that therapist-guided ICBT outperforms unguided interventions [42], as therapist engagement sustains participant adherence. For caregivers with limited digital literacy, technical assistance and age-friendly design features (eg, larger subtitles and enhanced visibility) are essential [43]. While video or telephone interventions enable direct communication and emotional support, they risk inefficiency in handling repetitive queries and may compromise continuity. WeChat public platforms or online websites could serve as supplementary tools. Telemedicine and mobile health solutions should be leveraged to comprehensively address caregivers’ diverse needs.

### Limitations

This review acknowledges several limitations. First, the included studies exhibited significant variability in intervention measures, participant characteristics, sample sizes, and outcome indicators, potentially limiting the generalizability of ICBT efficacy findings. Second, the lack of long-term follow-up assessments in most studies necessitates future research with extended evaluation periods to better understand sustained intervention effects. Third, while we only assessed risk of bias in RCTs, more comprehensive bias evaluations using standardized tools would strengthen future findings. Finally, the included studies originated from various countries, highlighting certain cultural differences. Nonetheless, this diversity underscores the feasibility and acceptability of ICBT among caregivers of patients with cancer.

### Implications for Future Research

To optimize resource use and enhance support for caregivers seeking online assistance, it is imperative to establish a dedicated support team composed of oncologists, nurses, and psychotherapists. Oncologists can build trust with patients and caregivers, promoting engagement and addressing maladaptive cognitions, which has been shown to improve outcomes and reduce attrition [44]. Nurses can serve as interdisciplinary coordinators and health educators, identifying caregiver needs, delivering symptom management guidance, and providing holistic biopsychosocial support. By fostering collaboration among multidisciplinary professionals, caregivers can receive more comprehensive support, capitalizing on the diverse strengths of various specialists and enhancing overall caregiver satisfaction.

### Conclusions

This study presents a scoping review of the application of ICBT among caregivers of patients with cancer. ICBT combines the advantages of CBT with the accessibility of smart devices. Preliminary evidence suggests that ICBT is effective in alleviating caregivers’ negative emotions, reducing stress, and enhancing positive experiences. Nevertheless, significant shortcomings remain in the research concerning intervention content, sample size, adherence, evaluation criteria, software development, and intervention teams. Currently, internet-based cognitive-behavioral interventions for caregivers of patients with cancer in our country are still in the nascent stages. It is crucial to draw upon relevant international studies while considering the unique characteristics of domestic caregivers to develop tailored ICBT intervention programs. By leveraging artificial intelligence to create safe and effective online platforms and fostering multidisciplinary collaboration, we can provide comprehensive physical and mental health interventions for caregivers, thereby genuinely alleviating their caregiving burden, reducing negative emotions, and enhancing their QoL.

## Supplementary material

10.2196/67131 Multimedia Appendix 1 Search strategies.

10.2196/67131 Checklist 1 Preferred Reporting Items for Systematic reviews and Meta-Analyses Extension for Scoping Reviews (PRISMA-ScR) checklist.

## Abbreviations

CBT: cognitive-behavioral therapy
CCO: cancer coping online
ICBT: internet-based cognitive behavioral therapy
PTGI: posttraumatic growth inventory
QoL: quality of life
RCT: randomized controlled trial
TSM: telephone-based symptom management
