# Supplementary material for: Internet-Based Cognitive Behavioral Therapy Interventions for Caregivers of Patients With Cancer: Scoping Review
Source: JMIR Cancer. 2025 Jun 4;11:e67131. doi: 10.2196/67131 (PMC12157961; doi:10.2196/67131)
Supplement: Multimedia Appendix 1 [file cancer-v11-e67131-s001.pdf]

Search Strategy for Wanfang Database
(limitation:the establishment of the database to June 6, 2024)

#1 ((主题=癌) OR 主题=癌症) OR 主题=肿瘤)
#2 (((主题=家人) OR 主题=配偶) OR 主题=照顾者) OR 主题=家属)
#3 #1 AND #2
#4 (((主题=网络) OR 主题=微信) OR 主题=互联网) OR 主题=移动)
#5 (((主题=认知行为疗法) OR 主题=认知疗法) OR 主题=行为疗法) OR 主题=认知行为)
#6 #4 AND #5
#7 #3 AND #6

Search Strategy for CNKI：
(limitation:the establishment of the database to June 6, 2024)

#1 （主题：癌症）OR（主题：癌）OR（主题：肿瘤）
#2 （主题：家人）OR（主题：配偶）OR（主题：家庭照顾者）OR（主题：照顾者）
#3 （主题：认知行为+认知行为疗法+认知行为干预）OR（主题：认知）OR（主题：行为）
#4 （主题：移动+移动互联网+移动通信）OR（主题：网络+网络化+基于网络）OR（主题：网络化认知行为+网络化认知行为疗法）
#5 #1 AND #2 AND #3 AND #4

Search Strategy for VIP：
(limitation:the establishment of the database to June , 2024)

（题名或关键词：肿瘤+tumors+tumour+瘤+癌症+癌）AND （题名或关键词：家属+family members+家庭成员+配偶+照顾者）AND （题名或关键词：行为疗法+behavioral therapy+行为治疗+认知行为+认知+cognitive）AND（题名或关键词：互联网+网络化认知行为+移动+移动通信）

Search Strategy for CBM：
(limitation:the establishment of the database to June , 2024)

#1 ((″癌″[常用字段] OR ″癌症″[常用字段] OR ″肿瘤″[常用字段])
#2 ((″家人″[常用字段] OR ″配偶″[常用字段] OR ″家属″[常用字段] OR ″照顾者″[常用字段] OR ″夫妻″[常用字段])
#3 #1 AND #2
#4 ((″网络″[常用字段] OR ″微信″[常用字段] OR ″移动″[常用字段] OR ″互联网″[常用字段])
#5 ((″认知行为″[常用字段] OR ″认知行为疗法″[常用字段] OR ″认知治疗″[常用字段] OR ″行为治疗″[常用字段] OR ″网络化认知行为″[常用字段])
#6 #4 AND #5
#7 #3 AND #6

Search Strategy for Web of Science :
(limitations:publication years:2024.6; document types:article; clinical trial;dissertation thesis)

#1 (((TS=(Neoplasms)) OR TS=(neoplas*)) OR TS=(carcinoma*)) OR TS=(Tumor)) OR TS=(oncology)) OR TS=(cancer)
#2 (((TS=(Caregivers)) OR TS=(caregiver*)) OR TS=(spouse)) OR TS=(family)) OR TS=(informal caregiver)) OR TS=(couple*)
#3 (((TS=(internet)) OR TS=(network)) OR TS=(online)) OR TS=(smartphone)) OR TS=(telephone)) OR TS=(computer)
#4 (((TS=(cognitive behavioral therapy)) OR TS=(cognitive behavio*)) OR TS=(behavio*therap*)) OR TS=(cognitive therap*)) OR TS=(ICBT)) OR TS=(CCBT)
#5 #1 AND #2 AND #3 AND #4

Search Strategy for Embase:
(limitations:date:2024.6.6;publication types: article;clicinal trial)

#1 'neoplasm'/exp
#2 'cancer*':ti,ab,kw OR 'tumor*':ti,ab,kw OR 'neoplas*':ti,ab,kw OR 'carcinoma*':ti,ab,kw OR 'oncolog*':ti,ab,kw
#3 #1 OR #3
#4 'caregiver'/exp
#5 'caregiver*':ti,ab,kw OR 'family':ti,ab,kw OR 'spouse*':ti,ab,kw OR 'relative*':ti,ab,kw OR 'informal caregiver':ti,ab,kw OR 'couple':ti,ab,kw
#6 #4 OR #5
#7 'internet'/exp
#8 'network':ti,ab,kw OR 'online':ti,ab,kw OR 'smartphone':ti,ab,kw OR 'computer':ti,ab,kw OR 'internet*':ti,ab,kw OR 'web*':ti,ab,kw OR 'mobile health':ti,ab,kw
#9 #7 OR #8
#10 'cognitive behavioral therapy'/exp
#11 'cognitive behavio*':ti,ab,kw OR 'behavio*therap*':ti,ab,kw OR 'cognitive therap*':ti,ab,kw OR 'ICBT':ti,ab,kw OR 'CCBT':ti,ab,kw
#12 #10 OR #11
#13 #3 AND #6 AND #9 AND #12

Search Strategy for CINAHL:
(limitations:date:2024.6;publication types: article;clicinal trial)

#1 (MH Neoplasms) OR (XB cancer*) OR (XB tumor*) OR (XB oncolog*) OR (XB malignan*) OR (XB neoplas*) OR (XB carcinoma*)
#2 (MH Caregivers) OR (XB caregiver*) OR XB (spouse) OR XB (family) OR XB (informal caregiver) OR XB (couple*)
#3 (MH Internet) OR XB (network) OR XB (online) OR XB (smartphone) OR XB (telephone) OR XB (computer)
#4 (MH cognitive behavioral therapy) OR XB (cognitive behavio*) OR XB (behavio* therap*) OR XB (cognitive therap*) OR XB (ICBT) OR XB (CCBT)

Search Strategy for Cochrane Library:
(limitations: date:2024.6.6;article type:trials)

#1 MeSH descriptor: [Neoplasms] explode all trees
#2 (cancer* OR tumor* OR oncolog* OR malignan* OR neoplas* OR carcinoma*):ti,ab,kw
#3 #1 OR #3
#4 MeSH descriptor: [Caregivers] explode all trees
#5 (caregiver* OR care* OR family OR spouse* OR relative* OR informal caregiver OR couple):ti,ab,kw
#6 #4 OR #5
#7 MeSH descriptor: [Internet] explode all trees
#8 (network OR online OR smartphone OR telephone OR computer OR internet* OR web* OR mobile health):ti,ab,kw
#9 #7 OR #8
#10 MeSH descriptor: [cognitive behavioral therapy] explode all trees
#11 (cognitive behavio* OR behavio*therap* OR cognitive therap* OR ICBT OR CCBT):ti,ab,kw
#12 #10 OR #11
#13 #3 AND #6 AND #9 AND #12

Search Strategy for pubmed:
(limitations: date:2024.6.6;article type:trials)

#1 Neoplasms[MeSH Terms]
#2 ((((neoplas*[Title/Abstract]) OR (carcinoma*[Title/Abstract])) OR (tumor[Title/Abstract])) OR (oncology[Title/Abstract])) OR (cancer*[Title])
#3 #1 OR #2
#4 Caregivers[MeSH Terms]
#5 ((((caregiver*[Title/Abstract]) OR (spouse[Title/Abstract])) OR (family[Title/Abstract])) OR (informal caregiver[Title/Abstract])) OR (couple*[Title/Abstract])
#6 #4 OR #5
#7 Internet[MeSH Terms]
#8 (((((network[Title/Abstract])) OR (online[Title/Abstract])) OR (smartphone[Title/Abstract])) OR (telephone[Title/Abstract])) OR (computer[Title/Abstract])
#9 #7 OR #8
#10 cognitive behavioral therapy[MeSH Terms]
#11 ((((cognitive behavio*[Title/Abstract]) OR (behavio*therap*[Title/Abstract])) OR (cognitive therap*[Title/Abstract])) OR (ICBT[Title/Abstract])) OR (CCBT[Title/Abstract])
#12 #10 OR #11
#13 #3 AND #6 AND #9 AND #12
