# Supplementary material for: Internet-Based Cognitive Behavioral Therapy Interventions for Caregivers of Patients With Cancer: Scoping Review
Source: JMIR Cancer. 2025 Jun 4;11:e67131. doi: 10.2196/67131 (PMC12157961; doi:10.2196/67131)
Supplement: Checklist 1 [file cancer-v11-e67131-s002.doc]

**Preferred Reporting Items for Systematic reviews and Meta-Analyses extension for Scoping Reviews (PRISMA-ScR) Checklist**

| **SECTION** | **ITEM** | **PRISMA-ScR CHECKLIST ITEM** | **REPORTED ON PAGE #** |
| --- | --- | --- | --- |
| **TITLE** | | | |
| Title | 1 | Internet-based Cognitive Behavioral Therapy Interventions for Caregivers of Cancer Patients：A Scoping Review |  |
| **ABSTRACT** | | | |
| Structured summary | 2 | **Background:** Cancer imposes significant physical and emotional distress not only on patients but also on their caregivers. In recent years, there has been a growing focus on the mental and physical well-being of caregivers. Among various psychological interventions, cognitive behavioral therapy (CBT) is widely recognized as one of the most effective approaches. However, traditional CBT is often limited by time and geographical constraints, resulting in delayed or inefficient support for caregivers. Internet-based cognitive behavioral therapy (ICBT) presents a valuable alternative for alleviating the caregiving burden and the negative emotions experienced by caregivers.  **Objectives:** (1) To provide a scoping review of ICBT interventions for cancer patient caregivers, examining intervention content, outcome measures, and effectiveness. (2) To offer insights and references for the development and clinical applications of ICBT programs tailored to cancer patient caregivers in China.  **Methods:** Relevant literature was systematically searched in PubMed, Web of Science, Cochrane Library, CINAHL, Embase, China National Knowledge Infrastructure (CNKI), Wanfang Data, and VIP Chinese Journal Database. The search timeframe was from database inception to June 6, 2024. Inclusion criteria encompassed intervention studies that implemented cognitive behavioral therapy for caregivers of cancer patients via the internet, WeChat, or mobile electronic devices. This category includes both randomized and non-randomized controlled trials. A total of 12 studies met the criteria and were included in the review.  **Results:** The intervention content included the following components: treatment initiation and brief introduction (n=5, 41%), cognitive education and restructuring (n=7, 58%), emotional expression and coping (n=6, 50%), cognitive restructuring and reinforcement (n=4, 33%), behavioral training and activation (n=9, 75%), problem-solving techniques (n=4, 33%), communication (n=5, 41%), and completion of treatment with follow-up consolidation (n=3, 25%). The intervention duration typically ranged from 6 to 8 weeks. Outcome indicators encompassed feasibility and acceptability, anxiety, depression, caregiver burden, and quality of life. ICBT demonstrated positive effects for caregivers of cancer patients. Most intervention programs were feasible and acceptable, with two out of five feasibility studies reporting recruitment rates below 50%. Attrition rates across studies ranged from 3% to 16%, and caregivers expressed satisfaction with the information, quality, and skills provided. ICBT exhibits a moderate effect in diminishing negative emotions among caregivers and alleviating caregiver stress. However, its impact on improving quality of life is not statistically significant, underscoring the need for long-term follow-up.  **Conclusions:** The implementation of ICBT for caregivers of cancer patients has demonstrated beneficial outcomes, attributed to its practicality and flexibility, which contribute to its greater acceptance among caregivers. Nevertheless, there is significant heterogeneity in intervention format, duration, and outcome indicators. It is necessary to develop optimal intervention strategies and secure online platforms based on the cultural background in China to improve the quality of life of caregivers. |  |
| **INTRODUCTION** | | | |
| Rationale | 3 | ICBT is an internet-based treatment approach that includes tools such as computers and mobile devices to deliver the fundamental content and skills of cognitive behavioral therapy by text, video, images, and audio |  |
| Objectives | 4 | there is significant heterogeneity in the forms of online interventions, intervention content, and outcome indicators. Thus, we performed a scoping review to analyze relevant literature and to identify existing issues in the research |  |
| **METHODS** | | | |
| Protocol and registration | 5 | This article has not been registered |  |
| Eligibility criteria | 6 | Inclusion criteria: (1) Study participants: Caregivers of confirmed (by pathology or imaging) patients with cancer, including offspring, parents, spouses, aged 18 years or older; (2) The intervention emphasizes the implementation of cognitive behavioral therapy via the internet, WeChat, mobile devices, or other applications; (3) Literature type: Original research, including randomized controlled trials or quasi-experimental studies; (4) Published literature in both Chinese and English.  Exclusion criteria: (1) Literature for which the full text could not be obtained; (2) Duplicated publications; (3) Conference abstracts; (4) Research protocols, reviews, and case studies. |  |
| Information sources* | 7 | CNKI, Wanfang Database, China Biomedical Literature Database, VP, PubMed, Web of Science, Embase, CINAHL, and Cochrane Library;The search timeframe extended from the establishment of the databases to June 6, 2024 |  |
| Search | 8 | 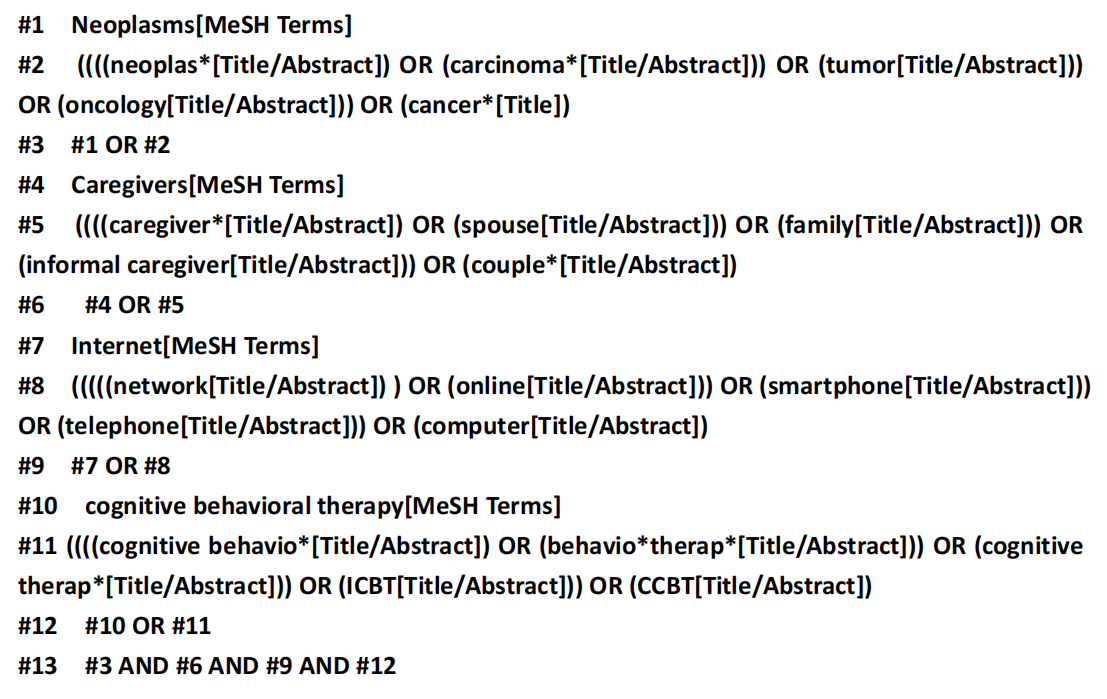 |  |
| Selection of sources of evidencet | 9 | Original experimental research |  |
| Data charting process‡ | 10 | Using standardized tables to independently extract data |  |
| Data items | 11 | We retrieved 1,005 articles; after removing duplicates, 878 articles remained. Following a thorough review of the titles, abstracts, and full texts, 12 articles were ultimately included in the study ; Figure 2 shows the specific screening process. Nine articles described a randomized controlled trial design, two articles documented a mixed-methods approach, and one article was classified as a quasi-experimental study. Regarding the study population, 7 articles focused on caregivers, and 5 articles targeted cancer patients and their caregivers. Table 1 details the basic characteristics of the included studies. |  |
| Critical appraisal of individual sources of evidence§ | 12 | The results of the literature search were imported into EndNote X9 for deduplication. Two researchers independently evaluated the titles, abstracts, and full texts of the articles according to the inclusion and exclusion criteria. In instances of disagreement, discussions were conducted with a third researcher to ascertain the final studies to be included |  |
| Synthesis of results | 13 | Data extraction included authors, publication year, country, study design, study population, sample size, intervention measures, and outcome indicators. |  |


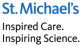

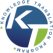


1


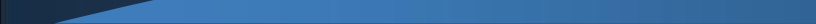


| **SECTION** | **ITEM** | **PRISMA-ScR CHECKLIST ITEM** | **REPORTED ON PAGE #** |
| --- | --- | --- | --- |
| **RESULTS** | | | |
| Selection of sources of evidence | 14 | 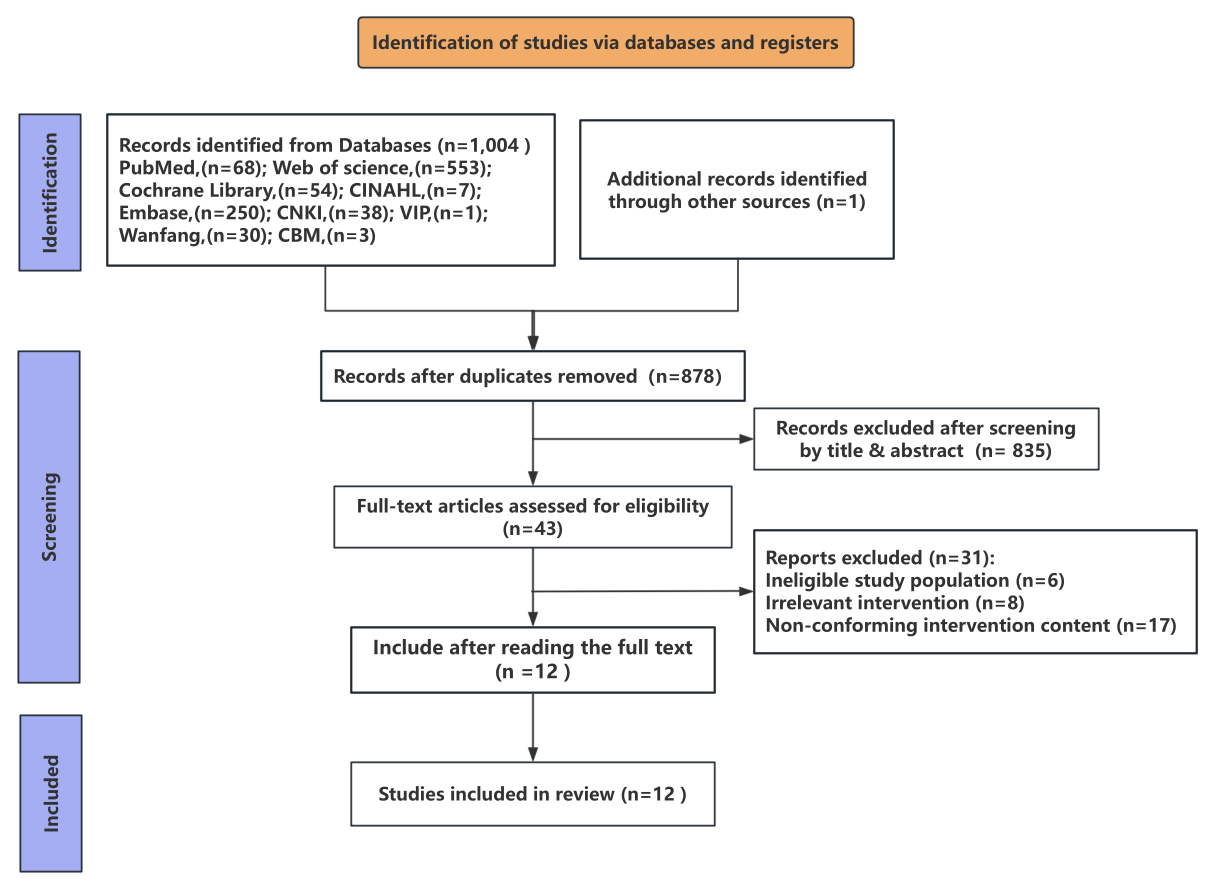 |  |
| Characteristics of sources of  evidence | 15 | Table 1. Basic characteristics of included literature |  |
| Critical appraisal within sources of evidence | 16 | — |  |
| Results of  individual sources of evidence | 17 | Table 1. Basic characteristics of included literature |  |
| Synthesis of results | 18 | (1) Feasibility. Feasibility was determined by recruitment, retention, and completion rates.Two studies had a recruitment rate of less than 50%, low participation.The primary factors contributing to suboptimal enrollment include aversion to online support, insufficient interest, demanding work schedules, and significant caregiving responsibilities. While the majority of participants successfully completed all intervention modules, attrition occurred in cases of elevated psychological distress, competing time commitments, or deterioration of the care recipient's health status . Across studies, attrition rates typically ranged from 3% to 16%, with one outlier reaching 31% . Qualitative analysis indicates this discontinuity pattern may reflect both the absence of personalized engagement in digital formats and intentional withdrawal upon achieving therapeutic goals.  (2) Acceptability. Acceptability was evaluated based on self-reported satisfaction with the intervention's content and participation activities. Many caregivers expressed positive feedback regarding the intervention, stating that “the online intervention is convenient, time-saving, and practical,” “they were satisfied with the information and quality provided,” “the skills learned were relevant to cancer treatment,” and “the intervention courses helped alleviate stress.” However, one study indicated that 33% of participants felt the intervention did not adequately address the needs of caregivers, while another study revealed disagreement about whether patients and caregivers should be treated together.  Specifically, 45% of caregivers preferred individual interventions, 36.4% favored some combined treatment, and 18.2% preferred fully integrated interventions.  (3) The impact on psychological outcomes. The cognitive-behavioral stress management program developed by Carr et al. enhanced caregiver awareness of adaptive stress management and coping relaxation exercises, thereby improving stress management during treatment. The results indicated a reduction in stress symptoms among caregivers, with a moderate effect size (r=0.39). Two additional studies demonstrated statistically significant differences in caregiver burden post-intervention (*P*<0.05). Caregivers often experience negative emotions such as anxiety, depression, or sadness due to prolonged caregiving and stress . Research suggests that ICBT has a moderate effect on reducing anxiety and depression. The structured writing intervention (‘Online-Trauertherapie’) implemented by Kaiser et al. for caregivers following cancer bereavement demonstrated significant intergroup interactions across various domains, including depression, anxiety, post-traumatic growth, and psychological well-being after the intervention, with effect sizes ranging from *d*=0.29 to 0.84. The study conducted by Trevino et al on the intervention MAC, grounded in cognitive behavioral therapy, revealed that changes in patient anxiety were positively correlated with changes in caregiver anxiety, whereas changes in depression and quality of life for both patients and caregivers were negatively correlated; however, these correlations were not statistically significant. Additionally, one study indicated that, although caregivers expressed satisfaction with the intervention content, the effects on improving participant anxiety and depression were not significant, potentially due to the low baseline levels of negative emotions among the participants. Another study indicated that the intervention did not improve the negative emotions of patients and caregivers, but further analysis revealed that increased guided imagery exercises reduced caregiver psychological distress .  (4) Quality of Life. Four studies demonstrated that the intervention significantly improved the quality of life for caregivers. In contrast, one study found that interventions specifically targeting spouses enhanced the quality of life for patients. However, three additional studies reported no significant improvement in the quality of life for caregivers. |  |
| **DISCUSSION** | | | |
| Summary of evidence | 19 | ①The Importance of Enhancing Caregiver Engagement ；②The Necessity of Establishing a Multidisciplinary Support Team；③ In-Depth Clinical Research on Networked Cognitive Behavioral Therapy for Caregivers |  |
| Limitations | 20 | The limited number of intervention articles on network cognitivebehavioral therapy for caregivers of cancer patients has led to insufficient comprehensive coverage for specific cancer types, resulting in inadequate targeting. |  |
| Conclusions | 21 | ICBT integrates the advantages of CBT and smart devices. Its effectiveness has been preliminarily validated in dispelling caregiver negative emotions, alleviating stress, and enhancing positive experiences. However, there are still significant shortcomings in terms of cohort size, adherence, evaluation criteria, software development, and intervention teams. Currently, Internet-based cognitive behavioral interventions for cancer patient caregivers are in their early stages in China. It is essential to draw on relevant international research while considering the characteristics of domestic caregivers to develop ICBT intervention programs. Furthermore, the integration of artificial intelligence can facilitate the creation of safe and effective websites, promoting multidisciplinary collaboration led by nurses to provide psychosocial interventions for caregivers, thereby genuinely alleviating their caregiving burden, reducing negative emotions, and improving their quality of life. |  |
| **FUNDING** | | | |
| Funding | 22 | No funding source |  |


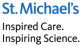

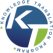


2
